# Circular RNA in Lung Cancer Research: Biogenesis, Functions, and Roles

**DOI:** 10.7150/ijbs.39212

**Published:** 2020-01-16

**Authors:** Congcong Zhang, Lifang Ma, Yongjie Niu, Zhixian Wang, Xin Xu, Yan Li, Yongchun Yu

**Affiliations:** 1 Shanghai Municipal Hospital of Traditional Chinese Medicine, Shanghai University of Traditional Chinese Medicine, Shanghai, 200071, P.R. China.; 2 Institute for Thoracic Oncology, Shanghai Chest Hospital, Shanghai Jiao Tong University, Shanghai, 200030, P.R. China.; 3 Department of Oncology, Shanghai Municipal Hospital of Traditional Chinese Medicine, Shanghai University of Traditional Chinese Medicine, Shanghai,200071, P.R. China.

**Keywords:** Lung cancer, circRNA, diagnostic biomarker, therapeutic targets

## Abstract

Lung cancer is one of the most common and deadly malignancies worldwide, in spite of advances in targeted therapy in recent years. An effective strategy for lung cancer prevention remains a major problem. Advances in next-generation sequencing have helped in understanding the RNA and identifying novel circular RNAs (circRNAs) that may have a broad impact on the early diagnosis and treatment of lung cancer. The circRNAs, exhibiting spatiotemporal-specific expression, are stable and conserved and present diverse biological functions in the normal and diseased states, including cancer. In this review, we summarize the recent advances in elucidating the functional role of circRNAs in lung cancer pathogenesis and discuss their potential mechanisms.

## Introduction

Lung cancer (LC) is the main cause of cancer mortality for both sexes globally. In 2018, 2.1 million new LC cases and 1.8 million deaths were reported worldwide, making LC the leading form of cancer in terms of both morbidity and mortality^1^, ^2^. Lung cancer has been pathologically classified into two main subsets: small cell lung cancer (SCLC), comprising 15% of the total cases^3^, and the rest 85% non-small cell lung cancer (NSCLC)^4^. The NSCLC is classified into three subtypes; large cell lung carcinoma (LCLC; 10%), lung squamous cell carcinoma (LUSC; 25%), and lung adenocarcinoma (LUAD; 40%). Recent advances in diagnostic and therapeutic procedures have resulted in a steady increase in the survival in most cancers, but not in LC whose 5-year survival rate is merely 17.7%^5^. The survival rate is considerably increased in patients with early-stage LC compared to patients with advanced LC. Therefore, it is crucial to establish the molecular mechanism underlying LC tumorigenesis and identify effective diagnostic biomarkers and therapeutic targets for LC.

The circRNAs are a class of covalently closed circular RNAs and ubiquitously exist in many species^6^. As a recently discovered form of endogenous non-coding RNA (ncRNA), circRNAs are typically generated from one to five exons 7 and are mainly located in the cytoplasm, with a small proportion of intron-containing circRNAs originating in the nucleus. Unlike linear RNAs, circRNAs exhibit the remarkable characteristic of being covalently closed continuous loop structure without 5′ to 3′ polarity and a polyadenylated tail, which makes them more stable in tissues and plasma^8^. Their unique structural properties suggest that these molecules are important transcripts, both physiologically and pathologically. Increasing studies have shown that circRNAs are expressed abnormally and act as an important regulatory element in the carcinogenesis and progression of LC, demonstrating that circRNAs can provide potential targets for the diagnosis and prognosis of LC. The present review briefly introduces current studies on the characteristics and biogenesis of circRNAs, summarizes their functional role and corresponding mechanisms in LC, and discusses the potential of circRNAs in the diagnosis and treatment.

## 2. History of circRNAs

In the 1970s^9^, Sanger et al. initially identified circRNAs in RNA viruses, like plant viruses. For a long time, due to the limitations of the detection techniques, most circRNAs were considered as the products of mis-splicing or by-products of pre-mRNA processing. In 2012, the abundance and ubiquity of circRNA in eukaryotes was recognized due to advances in RNA high-throughput sequencing and bioinformatics.

## 3. Biogenesis of circRNAs

**Figure 1:**
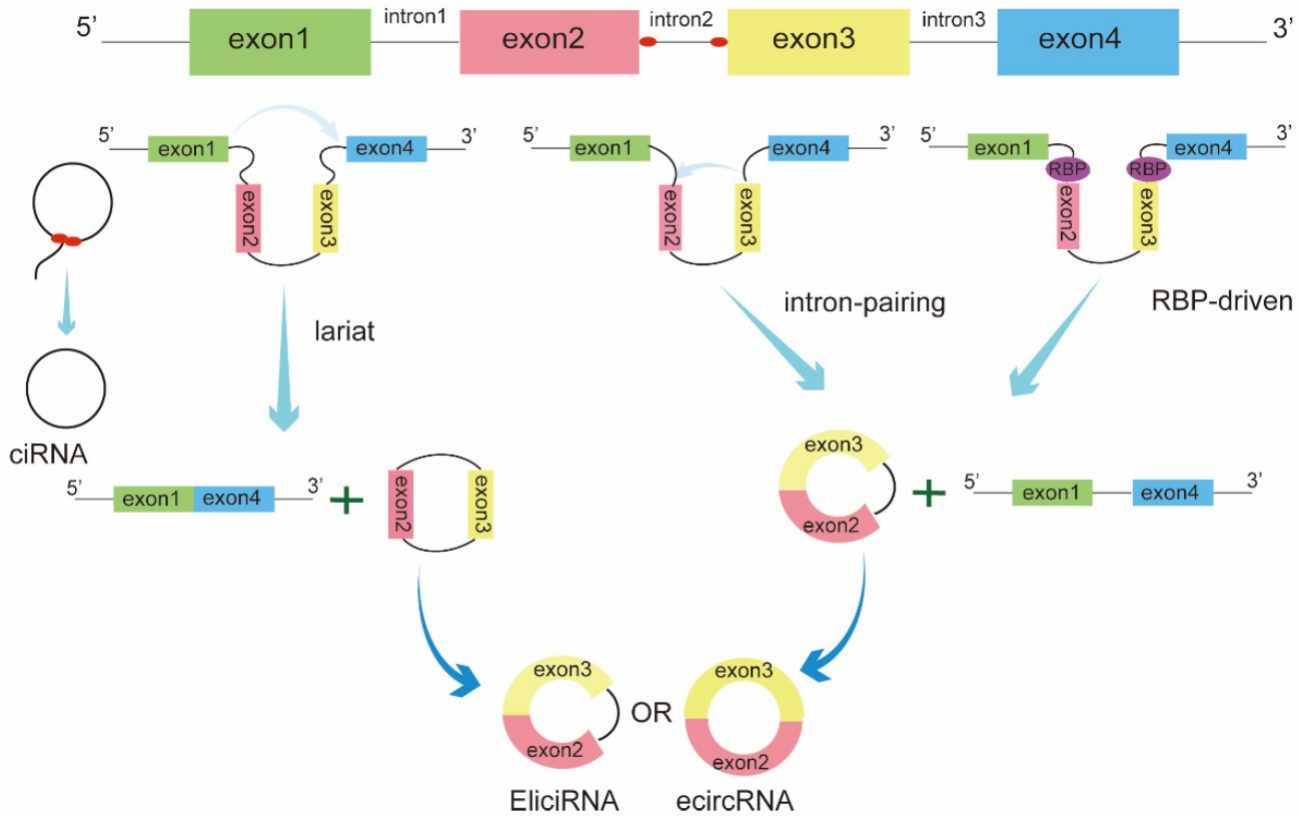
Biogenesis of circRNAs. The biogenesis of circRNAs is mainly regulated by three different mechanisms: lariat-driven circularization, intron pairing-driven circularization, and RNA-binding proteins (RBPs)-mediated circularization.

Based on the origin, circRNAs can be classified into three categories: exonic circRNAs (ecircRNAs), circular intron RNAs (ciRNAs), and exon-intron circRNAs (EIciRNAs) 10. The biogenesis of these circRNAs is regulated by different circularization mechanisms (Figure 1). Among these, ecircRNAs are the most abundant type, accounting for the majority of identified circRNAs^11^. Three main models of ecircRNA formation have been reported, namely, lariat-driven, intron-pairing-driven, and RNA-binding proteins(RBPs)-driven circularization. These models can be divided into two mechanisms, direct back-splicing and exon skipping. In the lariat-driven circularization model, a downstream 50 splice site of an exon joins an upstream 30 splice site, resulting in exon-skipping and the formation of an RNA lariat consisting of several exons and introns. The introns are then removed to generate ecircRNA^12^. In some cases, exons are circularized with introns that are retained between the exons, resulting in the generation of EIciRNAs. In addition, intron-pairing-driven and RBP-driven circularization events are direct back-splicing mechanisms. During this process, two flanking introns bracketing circularized exons are brought into close proximity by repeated ALU complementary elements, other complementary sequences without repetition, or RBPs^13^-^15^. Circular intron RNA biogenesis, which occurs via a lariat-derived mechanism, mainly depends on the conserved motifs at both ends of the intron, i.e., a 7-nt GU-rich element near the 5′ splice site and an 11-nt C-rich element near the branch-point site^16^.These motifs can prevent the intron from forming a circular debranching and cause the intron to form a circular structure. The resulting RNA circle is covalently linked through a 2′,5′ -phosphodiester bond at the junction site. The 3′ tail, stretching from the 3′ end of the intron to the branch point, is removed to produce stable ciRNAs.

## 4. Functions of circRNA

### 4.1. miRNA sponge

MicroRNAs (miRNAs) are important gene expression regulators, binding to specific sites of mRNA to prevent its translation or promote its degradation^17^. Increasing evidence has demonstrated that circRNAs can serve as competitive endogenous RNAs (ceRNAs) or miRNA sponges to inhibit miRNA function^18^, ^19^. In the pioneering study, a circRNA, named CDR1as (ciRS-7) was uncovered, which harbored >70 conserved miR-7 binding sites, and is a potent sponge of miR-7^20^. Moreover, Circ-ITCH is a circular RNA that could regulate the expression of ITCH by binding to multiple miRNAs^21^, ^22^.

### 4.2. Interaction with RBPs

The circRNAs can bind to RBPs and function as RBP sponges. The ciR-7/CDR1as was reported to correlate with Argonaute (AGO) proteins in a miR-7-dependent manner^18^. The RBPs, which was found in flies and humans, can bind to the muscleblind (MBL) protein at multiple binding sites and promote its circularization to form circMBL^14^. In addition, RBPs are involved in almost all cellular processes, including proliferation, apoptosis, and differentiation^23^.

### 4.3. Regulation of gene transcription

Circular RNAs are known to regulate gene transcription. Advanced studies have revealed that ciRNAs and EIciRNAs are primarily located in the nucleus and tend to function at the transcription level. The ciRNAs or EIciRNAs, like c-sirt7, are derived from lariats and can interact with the polymerase II complex, downregulating the expression of the corresponding genes ankyrin repeat domain 52 (ANKRD52) and sirtuin 7 (SIRT7)^24^. Thus, during the formation of circRNAs, it can modulate the expression of their parental genes, and this is one of the preconditions for the other functions of the circRNAs.

### 4.4. Translation to proteins

In addition to the non-coding functions of circRNAs, scientists recently confirmed that circRNAs could also be translated to proteins. As early as 1995, scientists found that the internal ribosome entry site (IRES) of the encephalomyocarditis virus played an important role in the translation of an artificial circRNA, indicating that circRNAs might have the potential to encode proteins^25^, ^26^. Legnini et al. found that circ-ZNF609, which functions in myogenesis, could translate proteins in murine myoblasts when driven by IRES, providing the first proof that endogenous circRNAs have translational capabilities^27^.

## 5. Functional roles of circRNAs in LC

The circRNAs are abnormally expressed and play an endogenous regulatory role in the carcinogenesis and development of LC. The expression and function of deregulated circRNAs are mentioned in Table 1 and Table 2, respectively.

### 5.1. Oncogenes

The extensively investigated circRNA, CDR1as, has been reported to be deregulated in a variety of cancers, including LC, and the first evidence of the function of circRNA as a microRNA sponge was the interaction between circular RNA ciRS-7 (CDR1as) and miRNA-7 (miR-7). The study showed a high expression of CDR1as in NSCLC tissues, which was strikingly associated with the high stage of tumor Node Metastasis(TNM); lymph node metastasis (LNM), and shorter overall survival (OS)^28^. Further, it was reported that CDR1as levels in tumor samples were negatively related to the expression of miR-7, which showed that the CDR1as might inhibit miR-7 in NSCLC. Overexpression of miR-7 restrained the gene expression of epidermal growth factor receptor (EGFR), CCNE1, and PIK3CD, which were restored by the overexpression of CDR1as. Moreover, CDR1as promoted cell growth via the miR-7/ EGFR/CCNE1/PIK3CD signals in NSCLC. These findings demonstrated that CDR1as functioned as an oncogene to inhibit the anti-tumor effects of tumor suppressor miR-7 by the up-regulation of proliferation index Ki-67, EGFR, CCNE1, and PIK3CD levels. The ErbB3 plays a major role in carcinogenesis and cancer progression. It was dramatically upregulated in LC, breast cancer, ovarian cancer, colorectal cancer, and gastric cancer^29^. High expression of ErbB3 correlated with tumor proliferation and migration in LC^30^ and miR-22 inhibited the progression of LUAD through post-transcriptional regulation of ErbB3^31^. Wang et al. indicated that hsa_circ_0012673 acted as a miR-22 sponge, and thus, regulated ErbB3 expression^32^. The hsa_circ_0012673 was found to be up-regulated in LUAD tissues versus adjacent tissue, and the expression level of hsa_circ_100338 was tightly associated with the tumor size. Furthermore, hsa_circ_0012673 could promote cell proliferation by sponging miR-22, which targeted ErbB3 in LUAD. The circMAN2B2 was reported to be highly expressed in the LC tissues^33^. The knockdown of circMAN2B2 repressed the proliferation and invasion of LC cells. Furthermore, the downregulation of circMAN2B2 enhanced the expression of miR-1275 in LC and inhibited the levels of its target, FOXK1. Taken together, circMAN2B2 functioned as an oncogene in LC through promoting FOXK1 expression by sponging miR-1275. The hsa_circRNA_103809 was overexpressed in LC tissues^34^, and its knockdown significantly inhibited the proliferation and invasion of LC cells and delayed tumor growth *in vivo*. The hsa_circRNA_103809 functioned as a sponge of miR-4302 by promoting the expression of ZNF121, thus enhancing the MYC protein levels in the LC cells. The MYC is a classical oncoprotein in various cancers, including LC, and an in-depth investigation demonstrated the significance of the hsa_circRNA_103809/miR-4302/ZNF121/MYC regulatory in LC progression. In summary, these findings illustrated that hsa_circRNA_103809 performed an oncogene function in LC and might be a potential therapeutic target. Qu et al. found a notable increase in the expression of hsa_circ_0020123 in cancer tissues compared to the normal lung tissues^35^. The upregulation of hsa_circ_0020123 was tightly associated with a poor differentiation degree, LNM, high TNM stage, and dismal prognosis in NSCLC patients. Overexpression of hsa_circ_0020123 significantly promoted tumor growth and metastasis in NSCLC cells, indicating that hsa_circ_0020123 could promote NSCLC progression by sponging miR-144. In addition, hsa_circ_0020123 could upregulate ZEB1 and EZH2 through competitively binding with miR-144. Finally, silencing hsa_circ_0020123 by siRNA delivery was shown to suppress NSCLC growth and metastasis *in vivo*. These findings suggested that hsa_circ_0020123-miR-144- ZEB1/EZH2 axis is critical for NSCLC progression, which indicates that hsa_circ_0020123 is a promising therapeutic for NSCLC treatment. Jiang et al. screened circRNA expression profiles and identified a total of 957 abnormally expressed circRNAs in NSCLC tissues compared to the normal tissues^36^. Studies have shown that hsa_circ_0007385 was significantly up-regulated in NSCLC tissue, and its knockdown suppressed the proliferation, migration, and invasion of NSCLC cells *in vitro*. Specifically, the downregulation of hsa_circ_0007385 enhanced the expression of miR-181. Thus, upregulated circRBM23 functioned as an oncogene in HCC through the downregulation of the tumor suppressor miR-181. Zhao et al. highlighted that circFADS2 expression was significantly upregulated in LC tissues and correlated with the advanced TNM stage, LNM, poor differentiation, and poor OS of NSCLC patients^37^. Further analysis indicated that circFADS2 knockdown inhibited the proliferation and invasion ability of the NSCLC cells. The circFADS2 could act as an oncogenic circRNA in LC progression by sponging miR-498. Taken together, these results suggested that circFADS2 could be a promising therapeutic target for LC treatment. The oncogenic fusion gene, Echinoderm Microtubule-associated protein-Like 4-Anaplastic Lymphoma Kinase (EML4-ALK), plays a vital role in the tumorigenesis of NSCLC. Tan et al. tested the EML4-ALK-positive cell line H2228 and found that the fusion-circRNA from EML4-ALK fusion gene (F-circEA) could promote cell migration and invasion^38^. Moreover, they indicated that another circRNA, F-circEA-2a, produced from EML4-ALK-v3b, is located in the cytoplasm and promoted cell migration and invasion^39^. The F-circEA-2a exists in the tumor, but not in the plasma of NSCLC patients with EML4-ALK fusion gene, further indicating that F-circEA has a significant diagnostic value for the diagnosis of EML4-ALK-positive NSCLC. Fusion-circRNA (F-circRNA) has been reported to have tumor-promoting properties *in vivo*, functioning as a diagnostic marker for the EML4/ALK1 gene fusion mutation in LC^40^. Multiple tumors are correlated with recurrent chromosomal translocations and the transcription of genes affected by chromosomal translocations could allow the formation of new circRNAs, namely F-circRNAs. Moreover, LC-associated EML4/ALK1 translocation originated from F-circRNAs, which are functionally relevant (tumor promoting), and represented a novel entry point for LC diagnosis. The circ_0026134 was highly expressed in lung tumors versus the normal adjacent tissue^41^. The knockdown of circ_0026134 significantly inhibited the proliferation and metastatic properties of NSCLC cells and induced cell apoptosis. Mechanistically, circ_0026134 acted as a sponge for miR-1256 and miR-1287, and thus, facilitates cell proliferation and invasion. Consequently, the overexpression of circ_0026134 acts as an oncogene in NSCLC cells. Zhou et al.^42^ showed that hsa_circ_0004015 was upregulated in NSCLC tissues and enhanced cell viability, proliferation, and invasion, whereas, its silencing exhibited opposite effects. The upregulation of hsa_circ_0004015 was tightly associated with the poor OS in NSCLC patients. Furthermore, hsa_circ_100338 served as a sponge for miR-1183 and induced the expression of the 3-phosphoinositide-dependent protein kinase 1 (PDPK) and stimulated AKT pathway. The circRNA, circAGFG1, was reported to have an oncogenic role in LC progression^43^. This result was obtained by real-time PCR analysis of 20 pairs of NSCLC tissues and their adjacent tissues. Subsequent experimental results proved that the silencing of circAGFG1 inhibited the proliferation and migration of A549 and H1299 cells. Mechanistic analyses demonstrated that circAGFG1 could act as a sponge for miR-203 to repress its effect on its target, ZNF281.

### 5.2. Tumor Suppressors

The circRNA-ITCH and hsa_circ_0043256 functioned as tumor suppressors in LC by up-regulating ITCH expression and inhibiting the activation of Wnt/β-catenin pathway; the expression of cir-ITCH was found to be decreased in LC^43^. As a circRNA, cir-ITCH could regulate the expression of ITCH by sponging miR-7 and miR-214, and consequently, inactivating LC cell proliferation. Further studies show that circ-ITCH inactivated Wnt/βcatenin pathway through transcriptionally regulated T-cell factor, β-catenin, c-Myc, and cyclinD1. The Wnt/β-catenin pathway was reported to play a critical role in tumorigenesis, and aberrant activation of the Wnt/β-catenin pathway was closely associated with NSCLC progression. Cinnamaldehyde (CA) has been reported to trigger NSCLC apoptosis through the inhibition of Wnt/β-catenin pathway in three kinds of NSCLC cells^44^. The expression of hsa_circ_0043256 was increased in NSCLC in response to CA treatment *in vitro* and *in vivo*. Overexpression of hsa_circ_0043256 could inhibit cell proliferation and induce apoptosis, whereas hsa_circ_0043256 knockdown resulted in the opposite effect. Moreover, hsa_circ_0043256 could function as a miR-1252 sponge, affecting the important negative regulator of the Wnt signaling pathway. These results suggested an important role of the hsa_circ_0043256/miR-1252/ITCH axis in the anti-tumor activity mediated by CA. The circ0006916 was down-regulated in LC cells and tissues. Overexpression of circ0006916 could affect the cell cycle distribution and inhibit cell proliferation^45^. Moreover, circ0006916 could act as a microRNA sponge in LC progression and exert regulatory functions by binding to miR-522-3p. These results indicate that circ0006916 may serve as a tumor suppressor in LC. The circRNA-FOXO3 may serve as a tumor suppressor in LC through sequestering miR-155 and enhancing FOXO3 expression^46^. Zhang et al. found that the expression of circRNA-FOXO3 was lower in NSCLC tissues and cell lines, and its upregulation significantly suppressed cell proliferation, migration, and invasion and promoted apoptosis. Mechanistically, circRNA-FOXO3 increases the expression of FOXO3 by sequestering miR-155, and thus, functions as a tumor-suppressor gene. Yang et al. investigated the expression and function of hsa_circ_0046264 in LC and reported its downregulation^47^. The overexpression of hsa_circ_0046264 significantly decreased the expression of miR-1245 and enhanced the levels of its target gene, BRCA2. Moreover, it has been found that hsa_circ_0046264 suppressed LC development and tumor growth *in vivo*. Liu et al. found that the expression of hsa_circ_0001946 was reduced in human lung cancer tissues and cell lines^48^. The knockdown of hsa_circ_0001946 promoted NSCLC cell viability, proliferation, migration, and invasion, as well as inhibition of cell apoptosis. More importantly, hsa_circ_0001946 downregulation activated the NER signaling pathway, which in turn reduced cisplatin sensitivity and increased DNA repair ability. These results confirmed that hsa_circ_0001946 not only performs an inhibitory function in NSCLC tumorigenesis but also might be a novel biomarker for predicting survival probability and the chemosensitivity of cisplatin in lung cancer patients. The expression of circPTPRA was dramatically downregulated in NSCLC tissues compared to the healthy lung tissues^49^. The downregulation of circPTPRA in NSCLC was associated with the clinicopathological features of NSCLC patients, including metastasis and shorter survival. In terms of mechanism, circPTPRA mediates its EMT-suppressive effects by sequestering miR-96-5p, which upregulates the downstream tumor suppressor Ras association domain-containing protein 8(RASSF8). These results advocated the circPTPRA/miR-96-5p/ RASSF8/Ecadherin axis as a new perspective on the role of circRNAs in NSCLC development.

### 5.3 Diagnostic value

Zhao et al. investigated the expression profile of circRNAs in early-stage lung adenocarcinoma tissues versus the normal tissues^50^. They identified a total of 357 circRNAs that were dysregulated in the early-stage lung adenocarcinoma, including 204 upregulated circRNAs and 152 downregulated circRNAs. Among these circRNAs, hsa_circRNA_404833 and hsa_circRNA_406483 displayed significant differences in their expression between LC tissues and normal tissues, which demonstrated that both might be exceptional potential candidates as the early diagnostic biomarker for LC. They also indicated that hsa_circRNA_404833 is a potential sponge of miR-149-5p, which inhibits cell motility and is involved in the acquired gefitinib resistance in LC. The hsa_circ_0000064 might act as a new promising biomarker and therapeutic target of LC^51^ as it was remarkably up-regulated in LC tissues and cell lines, which were related to the T stage, lymphatic metastasis, and TNM stage. The hsa_circ_0000064 knockdown suppressed cell proliferation, promoted cell apoptosis, and blocked cell cycle progression in A549 and H1229 cells. Western blot data further confirmed that the knockdown of hsa_circ_0000064 increased the level of the apoptosis-related Bcl-2 protein, while the expression of the cancer-related proteins involved in cell cycle, cell apoptosis, migration, and invasion was considerably restrained in the A549 and H1229 cells. This result indicated that hsa_circ_0000064 represented a promising biomarker for LC diagnosis. The circRNA_102231 might act as a potential therapeutic target for LC^52^. Zong et al. found that circRNA_102231 expression increases in LAC tissues and its high expression was closely implicated in the advanced TNM stage, LNM, and poor OS of LC patients. Function assays further confirmed that circRNA_102231 inhibition reduced the LC cells proliferation and invasive ability. Zhang et al. revealed the circRNA expression profiles in NSCLC tissues and their adjacent lung tissue and found that hsa_circ_0014130 (circPIP5K1A) expression was higher and its expression levels were associated with the TNM stage and lymphatic metastasis^53^. Gene oncology analysis and pathway analysis showed that hsa_circ_0014130 had a strong relationship with NSCLC development. More importantly, hsa_circ_0014130 might interact with five miRNAs and their corresponding mRNAs. Taken together, these studies revealed that hsa_circ_0014130 has a diagnosis value in distinguishing NSCLC from non-cancerous tissues. Gu et al. indicated that hsa_circ_0033155 was significantly downregulated in the NSCLC tissue and its dysregulation can be associated with lymphatic metastasis^54^. Furthermore, overexpression of hsa_circ_0033155 in NSCLC cells decreased cell proliferation, inhibits colony formation, and migration. Accordingly, hsa_circ_0033155 possesses a promising potential as a biomarker and a therapeutic target for NSCLC.

The circRNAs are insensitive to ribonucleases due to their stable circular structures, and thus, make them more stable and particularly attractive as liquid biopsy biomarkers. Recently, a global circRNA expression was detected using bioinformatics analysis^55^. The hsa_circ_0005962 was shown to be significantly upregulated in LUAD plasma and cells, whereas hsa_circ_0086414 was downregulated. The ROC curve analysis suggested that a signature comprising the two circRNAs had good diagnostic potential for LUAD. Moreover, high plasma hsa_circ_0086414 expression level was related to EGFR mutations. The hsa_circ_0005962 expression level was decreased after surgery in patients with LUAD, but hsa_circ_0086414 was not significantly different after surgery. Zhu et al. used a microarray technique for the detection of tumor-specific circRNA candidates in LAC tissue, and they found 39 upregulated and 20 downregulated circRNAs^56^. Among them, hsa_circ_0013958 was shown to be upregulated in all the LAC tissues, cells, and plasma. Mechanistically, they found that hsa_ circ_0013958 acted as a sponge for miR-134, and thus, up-regulated the oncogene CCND1, which plays a critical role in the development of NSCLC. Further analysis confirmed that hsa_circ_0013958 levels were correlated with TNM stage and lymphatic metastasis. These findings suggested that the expression level of hsa_circ_0013958 in stage I/II LAC patients was significantly up-regulated compared to the healthy controls, indicating that hsa_circ_0013958 could serve as a non-invasive biomarker, having high sensitivity and specificity, for the early detection and screening of LAC. A total of 185 differentially expressed circRNAs were identified in NSCLC tissues, including 120 upregulated circRNAs and 65 downregulated circRNAs^57^. The circFARSA was shown to be higher in cancerous tissues and was more abundant in patients' plasma than the controls. Functional analysis demonstrated that circFARSA overexpression promoted NSCLC cell migration and invasion. It might contribute to the development of LC by sponging several miRNAs, including miR-330- 5p and miR-326. These findings suggested that circFARSA was a noninvasive biomarker for NSCLC.

### 5.4 Prognostic value

Recently, circ_0067934 was reported to be overexpressed in NSCLC tissues and cell lines, and its elevated expression was significantly associated with the advanced TNM stage, lymph node status, distant metastasis^58^. Further study revealed that the circ_0067934 expression, which was related to a poorer OS, was used as a prognostic marker for invasive NSCLC. Moreover, circ_0067934 could regulate E2F7 as a miR-545 and miR-589 sponge. The expression level of circRNA_100876 was elevated in NSCLC tissues in the clinical samples^59^. High expression of circRNA_100876 was significantly correlated with LNM and advanced tumor staging in NSCLC. In addition, increased circRNA_100876 expression correlates with a short OS time in NSCLC patients. In conclusion, these findings demonstrated that circRNA_100876 is closely related to the carcinogenesis of NSCLC, and it could be used as a potential prognostic biomarker and therapeutic target for NSCLC. The circ_0016760 was highly expressed in NSCLC, and the upregulation of circ_0016760 was closely connected with advanced TNM stage, LNM, and adverse prognosis in NSCLC patients^60^. Moreover, the survival curve demonstrated that high levels of circ_0016760 were associated with shorter OS in NSCLC patients. Therefore, circ_0016760 might be a prognostic factor for NSCLC patients. Mechanistically, circ_0016760 acts as a sponge for miR-1287 and regulates GAGE1 expression. In summary, circ_0016760 might be involved in the tumorigenesis of NSCLC by circ_0016760/miR-1287/GAGE1 signaling. Xu et al. investigated the expression profile of circular RNA in LUSC. Among the differentially expressed circRNAs, hsa_circRNA_000122 was identified to be dramatically downregulated compared to the matched adjacent normal tissues, while hsa_circRNA_103827 was highly expressed^61^. The results indicated that the OS time of LSCC patients with high hsa_circRNA_103827 expression and low hsa_circRNA_000122 was significantly shorter. Consequently, has_circRNA-_103827 may be a promising biomarker for LC prognosis. Han et al. revealed that the circ-BANP expression, which was related to lower survival rate, increased in LC tissues^62^. Functionally, silencing circ-BANP inhibited cell proliferation and migration and induced invasion *in vitro* and delayed the progression of LC *in vivo*. Importantly, circ-BANP acts as a miR-503 sponge to upregulate the expression of LARP1. Collectively, the circ-BANP-mediated miR-503/LARP1 signaling performed a modulatory role in LC tumorigenesis, providing a promising prognostic indicator in LC. Li et al. found that hsa_circ_0000792 was significantly upregulated in LUAD compared with the matched noncancerous tissues^63^. The hsa_circ_0000792 was involved in signal transduction and cell communication in LUAD development. Further analysis showed that hsa_circ_0000729 expression had a negative correlation with miR-375. These findings supported that hsa_circ_0000792 holds great potential for LUAD diagnosis and prognosis. The circHIPK3 was found to be commonly expressed in six different NSCLC cell lines, with the highest expression in NCI-H2170 and the lowest in NCI-H1299^64^. The upregulation of circHIPK3 was able to stimulate cell proliferation in NCI-H2170. In contrast, interference with circHIPK3 expression was shown to inhibit NCI-H2170 cell proliferation. Moreover, circHIPK3 could sponge miR-379, which increased the levels of its target gene, IGF1. Thus, circHIPK3 performed a proliferation-promoting function in LC and could be a novel therapeutic target for NSCLC. Chen et al.^65^ also confirmed that the knockdown of circHIPK3 significantly suppressed NSCLC cell proliferation, migration, and invasion and induced macroautophagy/autophagy. The circHIPK3 silencing was shown to inhibit cell growth and might be partially associated with the induction of autophagy in a subset of LC cell lines. Further study indicated that circHIPK3 abrogation induced autophagy via STAT3-PRKAA and was STK11-dependent. It functions as an oncogene and autophagy regulator, and might be a potential prognostic indicator and therapeutic target in LC. Loss-of-function studies indicated that circUBAP2 knockdown suppressed the proliferation and invasion of lung adenocarcinoma^66^. *In vitro* study showed that circUBAP2 silencing inhibited the expression of CDK6, cyclin D1, c-IAP1, Bcl-2, SuUBRrvivin, FAK, Rac1, and MMP2, while the expression of p27 and Bax was significantly increased. Moreover, circUBAP2 knockdown can suppress the activities of Rac1 and FAK. Consequently, circUBAP2 plays an important role in the proliferation and invasion of human LC and is a promising target for the treatment of NSCLC. Ding et al. compared the expression of circ_001569 between paired NSCLC and adjacent normal tissues^67^. The result indicated that the expression of circ_001569 was increased in NSCLC tissues. The upregulation of circ_001569 in NSCLC was strikingly associated with tumor differentiation, LNM, and TNM classification. Functional assay results indicated that circ_001569 knockdown suppressed the proliferation of NSCLC *in vitro*. In addition, circ_001569 knockdown could inhibit the Wnt/β-catenin signaling pathway in NSCLC cells. Therefore, the high expression of circ_001569 might present a risk factor for predicting the prognosis of patients with NSCLC. Li et al. proposed that hsa_circ_0079530 was upregulated in LC tissues and tightly associated with tumor size and LNM^68^. The hsa_circ_0075930 knockdown suppressed the proliferation and also induced the migratory and invasive capabilities of NSCLC cells by reversing epithelial-mesenchymal transition (EMT), which is associated with a more invasive phenotype in tumors. This data suggests that hsa_circ_0079530 performed an oncogenic role in NSCLC and may serve as a prognosis predictor and therapeutic target for NSCLC. Liu et al.^69^ found that the circ-FOXM1 expression, which was closely associated with lymph node invasion, higher TNM stage, and unfavorable prognosis, was upregulated in the lung cancer tissues. The circ-FOXM1 promoted the proliferation and invasion of NSCLC cells by upregulating the levels of PPDPF and MACC1 through sponging miR-1304-5p. This finding indicated that circ-FOXM1/miR-1304-5p/PPDPF/MACC1 signaling is essential for the development and progression of NSCLC.

## Conclusions

Lung cancer is the root cause of cancer-associated deaths globally. Considering the high morbidity and mortality of LC, many trials are in progress, looking for more advanced therapeutic approaches. Targeted therapy is now one of the important hot spots of cancer treatment research; circular RNA is a new frontier in cancer research. It has been indicated that altered circRNA expression can affect the tumorigenesis and progression of LC, and these circRNAs hold important clinical relevance due to their advantages in LC diagnosis, therapy, and prognosis. Circular RNAs possess multiple advantages, including relatively long and stable structures, suggesting that circRNAs are ideal diagnostic biomarkers and promising therapeutic targets for LC, which could provide a novel avenue for us to develop a panel of specific circRNAs for different types of LCs that can be safely and successfully integrated into clinical practice. Recent studies have found that circRNAs function as miRNA sponges, regulate transcription, or affect gene/protein expression involved in the cell cycle, proliferation, invasion, and metastasis. However, compared with the other ncRNAs, the study of circRNAs in LC is still in the early stage, and only a small number of functional circRNAs have been discovered and characterized. Circular RNAs have a variety of important biological functions, but the existing research is mostly devoted to the miRNA sponge function of the circRNAs, which makes the related research not comprehensive or deep enough; other functions of circRNA require further study. The functional mechanism of the circular RNAs in LC is not limited to miRNA sponges; several studies have unveiled the other functions of circRNAs. The complex regulatory network of the competing endogenous RNA (ceRNA) needs to be enriched by incorporating more factors. The improvement in the second-generation sequencing technology and the reduction in cost, along with the establishment of an unbiased bioinformatic method will lead to the identification of a large number of novel circRNAs, whose potential mechanisms would be studies, which will accelerate the clinical application of circRNAs in LC diagnosis and therapy.

## Figures and Tables

**Figure 2:**
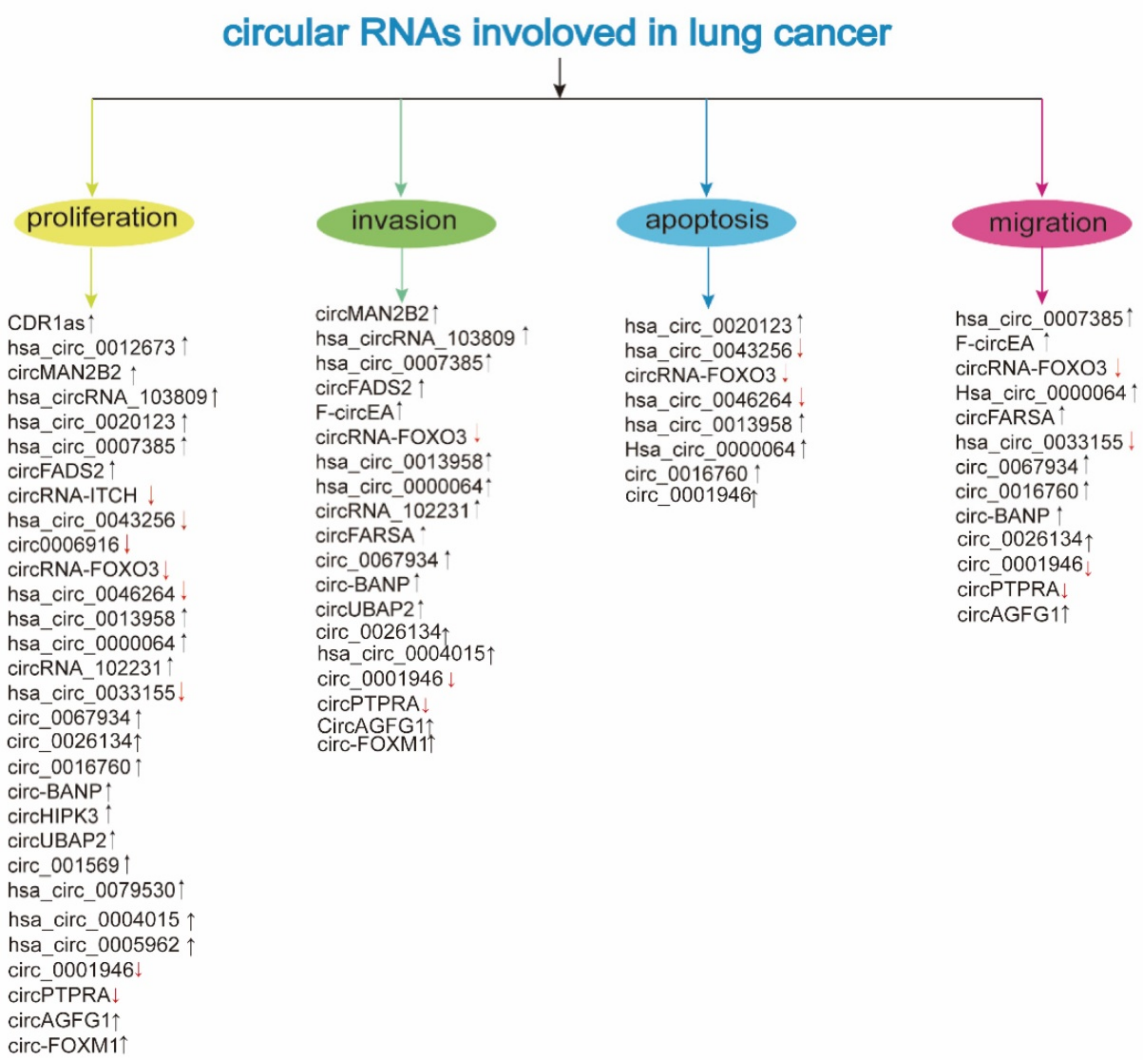
Involvement of circRNAs in lung cancer (↓: downregulation; ↑: upregulation; see the text for further details)

**Table 1:** Upregulated circRNAs in lung cancer (LC)

CircRNA	Expression Change	Function	Clinical relevance	Possible Mechanism	Reference
CDR1as	Up	proliferation(+)	TNM LNM OS	miRNA sponge	28
hsa_circ_0012673	Up	proliferation(+)	tumour size	miRNA sponge	32
circMAN2B2	Up	proliferation(+) invasion(+)	Unknown	circMAN2B2/miR-1275/FOXK1 signaling	33
hsa_circRNA_103809	Up	proliferation(+) invasion(+)	Unknown	hsa_circRNA_103809/miR-4302/ZNF121/MYC	34
hsa_circ_0020123	Up	Proliferation(+) apoptosis(-)	TNM LNM	hsa_circ_0020123-miR-144- ZEB1/EZH2	35
hsa_circ_0007385	Up	Proliferation(+) migration(+) invasion(+)	tumor growth	miRNA sponge	36
circFADS2	Up	proliferation(+) invasion(+)	TNM LNM OS	miRNA sponge	37
F-circEA	Up	migration(+) invasion(+)	Unknown	Not mentioned	38, 39
circ_0026134	Up	proliferation(+) Migration(+) invasion(+)	Unknown	miRNA sponge	41
hsa_circ_0004015	Up	proliferation(+) invasion(+)	OS	circ_0016760/miR-1183/PDPK1	42
circAGFG1	Up	proliferation(+) Migration(+) EMT(+)	Unknown	Not mentioned	43
Hsa_circ_0000064	Up	proliferation(+) migration(+) invasion(+) apoptosis(-)	T stage TNM LNM	Not mentioned	51
circRNA_102231	Up	proliferation(+) invasion(+)	TNM LNM OS	Not mentioned	52
hsa_circ_0005962	Up	proliferation(+)	Unknown	Not mentioned	55
hsa_circ_0014130	Up	Not mentioned	TNM LNM	miRNA sponge	53
circFARSA	Up	migration(+) invasion(+)	Unknown	miRNA sponge	57
circ_0067934	Up	proliferation(+) migration(+) invasion(+) EMT(+)	TNM LNM OS	miRNA sponge	58
circRNA_100876	Up	Not mentioned	LNM tumor staging OS	miRNA sponge	59
circ_0016760	Up	proliferation(+) migration(+) apoptosis(-)	TNM LNM	circ_0016760/miR-1287/GAGE1	60
circ-BANP	Up	proliferation(+) invasion(+) migration(+)	OS	circ-BANP-mediated miR-503/LARP1	62
hsa_circ_0000792	Up	Not mentioned	TNM	miRNA sponge	63
circHIPK3	Up	proliferation(+) migration(+) invasion(+)	Unknown	miRNA sponge	64, 65
circUBAP2	Up	proliferation(+) invasion(+)	Unknown	miRNA sponge	66
circ_001569	Up	proliferation(+)	LNM TNM OS	Wnt/β-catenin	67
hsa_circ_0079530	Up	Proliferation(+) migration(+) invasion(+) EMT(+)	LNM tumor size	Not mentioned	68
circ-FOXM1	Up	proliferation(+) invasion(+)	LNM TNM	circ-FOXM1/miR-1304-5p/PPDPF/MACC1 signaling	

**Table 2:** Downregulated circRNAs in lung cancer (LC)

CircRNA	Expression Change	Function	Clinical relevance	Possible Mechanism	Reference
circRNA-ITCH	down	proliferation(-)	Unknown	Wnt/β-catenin	43
hsa_circ_0043256	down	proliferation(-) apoptosis(+)	Unknown	Wnt/β-catenin	44
circ0006916	down	proliferation(-)	Unknown	miRNA sponge	45
circRNA-FOXO3	down	proliferation(-) migration(-) invasion(-) apoptosis(+)	Unknown	miRNA sponge	46
hsa_circ_0046264	down	proliferation(-) apoptosis(+)	Unknown	Wnt/beta-catenin	47
circ_0001946	down	proliferation(-) migration(-) invasion(-) apoptosis(+)	Unknown	Nucleotide Excision Repair Signaling	48
circPTPRA	down	proliferation(-) migration(-) invasion(-) EMT(-)	metastasis OS	circPTPRA/miR-96-5p/RASSF8/E-cadherin	49
hsa_circ_0033155	down	proliferation(-) migration(-) PTEN(+)	LNM	Not mentioned	54
circ_0086414	down	Not mentioned	Unknown	Not mentioned	55

## Abbreviations

LC: Lung cancer
circRNAs: circular RNAs
SCLC: small cell lung cancer
NSCLC: non-small cell lung cancer
LCLC: large cell lung carcinoma
LUSC: squamous cell carcinoma
LUAD: lung adenocarcinoma
ecircRNAs: exonic circRNAs
ciRNAs: circular intron RNAs
EIciRNAs: exon-intron circRNAs
ceRNAs: competitive endogenous RNAs
RBP: RNA-binding proteins
MBL: muscleblind
AGO: Argonaute
ANKRD52: ankyrin repeat domain 52
SIRT7: sirtuin 7
IRES: internal ribosome entry site
miR-7: miRNA-7
TNM: Tumor Node Metastasis
LNM: lymph node metastasis
OS: overall survival
CA: Cinnamaldehyde
EGFR: epidermal growth factor receptor
EML4-ALK: Echinoderm Microtubule-associated protein-Like 4-Anaplastic Lymphoma Kinase
F-circEA: Fusion-circRNA from EML4-ALK
F-circRNA: Fusion-circRNA
PDPK: 3-phosphoinositide-dependent protein kinase 1
RASSF8: Ras association domain-containing protein 8
EMT: epithelial-mesenchymal transition
PPDPF: pancreatic progenitor cell differentiation and proliferation factor
MACC1: metastasis-associated in colon cancer 1
